# Fluorescence Molecular Painting of Enveloped Viruses

**DOI:** 10.1007/s12033-012-9616-6

**Published:** 2012-10-28

**Authors:** Christoph Metzner, Feliks Kochan, John A. Dangerfield

**Affiliations:** 1 Institute of Virology, University of Veterinary Medicine, Vienna, Veterinarplatz 1, 1210 Vienna, Austria; 2 Anovasia Pte Ltd, 20 Biopolis Way, #05-518 Centros, Singapore, 138668 Singapore

**Keywords:** Molecular painting, GPI-anchored protein, Surface modification, Lentivirus, Herpesvirus, HIV, Influenza, Fluorescence labeling, Viral attachment, Gene therapy

## Abstract

In this study, we describe a versatile, flexible, and quick method to label different families of enveloped viruses with glycosylphosphatidylinositol-modified green fluorescent protein, termed fluorescence molecular painting (FMP). As an example for a potential application, we investigated virus attachment by means of flow cytometry to determine if viral binding behavior may be analyzed after FMP of enveloped viruses. Virus attachment was inhibited by using either dextran sulfate or by blocking attachment sites with virus pre-treatment. Results from the FMP–flow cytometry approach were verified by immunoblotting and enzyme-linked immunosorbent assay. Since the modification strategy is applicable to a broad range of proteins and viruses, variations of this method may be useful in a range of research and applied applications from bio-distribution studies to vaccine development and targeted infection for gene delivery.

## Introduction

Modification of viral surfaces has a number of potential applications, both in applied and basic molecular biotechnology. From a technological angle, mainly two fields are concerned: gene therapy and vaccination strategies. Tagging of viral surfaces with reporter molecules or affinity tags such as GFP or histidine repeats may facilitate simple purification and concentration of viruses and viral vectors, as well as allow following the fate of the particles more easily, e.g., to aid imaging or collection from specific compartments in bio-distribution studies. Additionally, binding proteins (e.g., growth factors, adhesion molecules, and single chain antibodies) presented on viral surfaces may be useful to redirect infection to specific subsets of cells in gene therapy allowing infection targeting for gene therapy [1, 2], a challenging, yet potentially rewarding approach. Finally, the display of immunologically relevant molecules (i.e., cytokines and regulators of complement activity) may help to suppress or stimulate immune response studies in gene therapy [3, 4] or vaccination approaches [5].

Currently such modifications are introduced most often by genetic engineering of the virus producing cells. However, this process is time consuming, lacks flexibility and control, and may not be applicable in certain cases, i.e., when the virus cannot be produced in cell culture, when sufficient knowledge about the molecular biology and/or genetics of the virus is not available in order to carry out the genetic modification, or when pre-existing manufacturing processes are already implemented in industry. Methods such as fluorescence molecular painting (FMP) which modify viruses after they have left the producing cells (post-exit) circumvent the problems of transfection-based approaches mentioned above and could also reduce the time and costs for new product development and validation processes since a single agent is added (which can be manufactured to high standards) rather than an entire new biological entity.

*Post*-*exit* methods of virus surface modification fall into three categories: (i) direct (covalent) chemical modification [6–8], (ii) using adaptor systems such as streptavidin/biotin [9–11], or (iii) employing agents which associate or insert into lipid membranes [12–15]. While the issue of bio-compatibility is often problematic when using a covalent modification approach, the added level of complexity introduced by adaptors may complicate experimental procedures. While the first two approaches usually involve engagement of pre-existing proteins on the virus surface, which may change properties of the viral particles considerably, the last introduces a novel agent (which usually leaves only a small “footprint” on virus surfaces) into fully formed viral particles, thus giving less opportunity for functional disturbances. Obviously, the last approach is only applicable to enveloped viruses. Artificial lipid-targeting structures may be used [14, 15] as well as naturally occurring ones [12, 13, 16]. We have used such a membrane association-based strategy to label a range of virus species, i.e., lenti-, herpes-, and orthomyxovirus (LV, HV, and OM, respectively), with a fluorescent marker protein carrying a specific post-translational modification. In all eukaryotic cells, depending on the cell type, ~0.5 % of proteins are modified with a glycosylphosphatidylinositol (GPI) moiety [17]. The function of these residues is to anchor the proteins to the outer surface of the cell membrane and possibly also target them to distinct micro-domains within the membrane [13, 18]. Presence of a GPI anchor can be engineered onto any protein of choice by encoding a fusion of the GPI-signaling sequence (GSS) into the sequence by molecular biology means [18–21]. Once present at the amino acid level in the endoplasmatic reticulum, the GSS allows the transamidase enzyme complex to add a GPI moiety as a post-translational modification step.

An interesting feature of GPI proteins is that once purified from cells, the proteins can re-insert into lipid bilayer membranes [20–22]. While this phenomenon known as “painting” or “cell painting” has been described for cells already in the 1980s [22], it was only recently applied to retroviral envelopes [12] where it was called “virus painting” or more generally for all membranes “molecular painting” (MP). In the latter carried out in our group, the GPI-anchored human complement regulatory protein CD59 (Protectin) was introduced to the envelopes of retro- and lentiviral gene therapy vectors. This insertion of the protein itself did not reduce infectivity of the viral vectors [12]. The calculated numbers of inserted molecules (roughly estimated at 150) were in the range of viral surface glycoprotein amounts, thus suggesting a potential biological relevance of the inserted protein. In this study, we show for the first time that a novel function (the fluorescence) could be transferred onto the viral particle. We constructed, expressed, and purified two different variants of green fluorescent protein (GFP) containing a 6× histidine tag to allow metal ion affinity purification and the GSS from CD55 (or decay accelerating factor DAF, another regulator of complement activity). We investigated the insertion behavior of both variants, as well as the insertion behavior in the absence of a GPI anchor. We could also successfully attach two different GPI-anchored proteins (GPI-AP) to viral vectors simultaneously. Finally, we used this system to determine the influence of inhibitory agents on viral attachment in a flow cytometry-based assay.

## Materials and Methods

### Plasmids and Cells

Generation of pCD59hisneo and expressing cells was described previously [12]. A similar approach was followed to introduce the 6× his tag and generate pGPI-EHhyg and pMonoGGhishyg based on constructs pGFP-GPI(DAF) [18] provided as a kind gift by the group of Daniel Legler at the Biotechnologie Institut Thurgau, Switzerland, containing the original GFP sequence from pEGFP-C1 (Clontech, Palo Alto, CA) and pJB20-GPI-GFPmutA206 provided as a kind gift by the group of Gerhard Schütz at Johannes-Kepler-University in Linz, Austria, containing the original monomeric GFP sequence described previously [23]. In brief, a 2-step PCR mutagenesis protocol was used for re-cloning into the pcDNA3.1hyg(+) vector (Invitrogen) using the following primers in first step PCR, generating two separate fragments: EGHindIIIF (5′-CGCGCGCAAGCTTAATCAAAACATGG-3′) and HisEG3 (5′-GTGGTGGTGATGGTGGTGCTTGTACAGCTCGTCCATGCCGAGAGT-3′) for the first fragment of pGPI-EHhyg; MEHindIIIF (5′-CGCGCGCAAGCTTAATCAAAACATGGCTCAGCGGATGACA-3′) and MonoHisEG3R (5′-GTGGTGGTGATGGTGGTGCTTGTACAGCTCGTCCATGCCGAGAGT-3′) for the first fragment of pMonoGGhishyg. HisEG1F (5′-CACCACCATCACCACCACCCAAATAAAGGAAGTGGAACC-3′) and EGApaIR (5′-GAATAGGGCCCTAATCAGCAAGCCCATG-3′) were used to generate the second fragments in both cases. The two primary fragments were joined in the 2nd step PCR using the outer primers (EGFHindIIIF–EGApaIR and MEHindIIIF–EGApaIR, respectively). Resulting fragments were cloned into the vector backbone using HindIII and ApaI sites. Expressing cell populations were derived from parental CrFK/HEK293 cells by lipofection using TurboFect reagent (Fermentas), according to manufacturer’s instructions, thus creating CrFKpGPI-EHhyg and HEK293 monoGGhishyg. STAR (ECACC-No. 04072119) and STAR-A (ECACC-No. 04072119) were used with the kind permission of Prof. M Collins. MDCK (CCL-34), CrFK (CCL-94), HEK293 (CRL-1573), and Hela (CCL-2) were obtained from ATCC. All cells were cultured in DMEM supplemented with 10 % FCS containing the appropriate antibiotics, with the exception of MDCK cells which were cultured in 50 % DMEM/50 % Ham’s F12 supplemented with 10 % FCS and 2 mM l-glutamine.

### PI-PLC Treatment

Twenty T175 flasks containing cells expressing monoGGhis were harvested and split into two aliquots. One was directly processed for FPLC, while the other was incubated for 2 h at 30 °C under occasional inversion with 5 U of *B. subtilis* PI-PLC (Sigma-Aldrich) in a total volume of 5 ml. After centrifugation for 10 min at 1,500 rpm (440×*g*), the supernatant from treated cells was filled up with FPLC sample application buffer (50 mM Tris–HCl, 50 mM NaCl, 35 mM Imidazole, 1 % NP40 octylglucoside, pH 7.4) to a total volume of 25 ml and used for subsequent FPLC.

### Protein Purification

Purification procedures were carried out as described previously [12]. In brief, 4–6 confluent T175 flasks of GPI-AP expressing cells were harvested by scraping after washing cells with 10 ml PBS. Cells were scraped into a total of 25 ml sample application buffer (50 mM Tris–HCl, 50 mM NaCl, 35 mM Imidazole, 1 % octylglucoside, pH 7.4). 80 μl of protease inhibitor complex (Sigma-Aldrich) was added. Samples were incubated for at least 30 min on ice before centrifugation for 30 min at 2,400×*g* at 4 °C. Samples were filtered through 0.45 μm filters (Sarstedt) before application to a ÄktaPrime plus FPLC device (GE Healthcare). Prepacked 5 ml HisTrap FF Crude columns (GE HealthCare) were used. The columns were washed using washing buffer (50 mM Tris–HCl, 50 mM NaCl, 35 mM Imidazole, pH 7.4), and elution was achieved by using elution buffer (50 mM Tris–HCl, 50 mM NaCl, 600 mM Imidazole, pH 7.4). Fractions were collected during elution. Presence of GPI-anchored variants of GFP in fractions was determined by immunoblotting. Positive fractions were pooled and concentrated by ultrafiltration using Amicon Ultra filter devices (Millipore, 5 and 10 kDa molecular weight cut-off) and washed twice with protein storage buffer (PSB, 50 mM Nacl, 50 mM Tris–HCl, pH 7.4). Concentration of protein was determined using a modified Lowry assay (BioRAD Protein Dc kit), according to manufacturer’s instructions.

### Virus Production and Harvesting

The stably virus-producing cell line STAR-A was used for making lentiviral particles pseudotyped with amphotropic 4070A MLV env. CrFK cells were used to produce wild-type Feline herpes virus 1 (FHV-1) particles. STAR-A and CrFK cells were cultured in DMEM supplemented with 10 % FCS. Approximately 72 h prior to harvesting, culture medium was replaced with DMEM without FCS. At the same time, CrFK cells were infected with FHV by diluting virus stock 1:100. For stably producing STAR-A, no infection was necessary. MDCK cells were used to generate Influenza A/Aichi/2/68 (H2N3) particles. The initial Influenza viral stock was a kind gift from Andrea Wolkerstorfer (SAVIRA Pharmaceutical, Vienna, Austria). MDCK cells were cultured in 50 % DMEM/50 % Ham’s F12 supplemented with 2 mM l-glutamine and 10 % FCS. Approximately 72 h prior to harvesting, culture medium was replaced with 50 % DMEM/50 % Ham’s F12 supplemented with 2 mM l-glutamine and 5 μg/ml trypsine. MDCK cells were infected with Influenza by diluting virus stock 1:100. For all viruses, supernatants from either 4 (LV) or 2 (HV, OM) T-175 flasks per sample were collected and purified as follows: a 10-min centrifugation step at 2,400×*g* was followed by filtration of the supernatant through 0.45 μm filters. Finally, supernatants were ultracentrifuged for 2 h at 21,000 revolutions per min (equivalent to an average rotational centrifugal force of approximately 54,000×*g*) in a Beckman XL-70 ultracentrifuge using a SW32Ti rotor. Samples were re-suspended in an appropriate volume of DMEM.

### Product-Enhanced Reverse Transcriptase Assay (PERT)

Product-enhanced reverse transcriptase assay was carried out as previously described [24]. In brief, 20 μl of concentrated supernatants from virus producer cells was mixed with an equal amount of disruption buffer (40 mM Tris–Cl, pH 8.1; 50 mM KCl; 20 mM dithiothreitol-DTT; 0.2 % Triton X-100) and incubated at room temperature for 2 min to lyse the virions. Starting from this dilution, 1:100 and 1:1,000 dilutions were made from each sample. As a standard, a serial dilution of purified MoMLV RT (Promega) in RT-dilution buffer (20 mM Tris–Cl, pH 7.5; 50 mM KCl; 0.25 mM EDTA, pH 8.0; 0.025 % Triton X-100; 50 % glycerol; 0.2 mM DTT) was used. In the reverse transcription step of the assay, 10 μl of either standard, sample, or negative controls was incubated with 20 ng of MS2 bacteriophage RNA for 1 h at 37 C. MS2 DNA, generated during the RT-step, was quantified by real-time PCR. PCR was performed with a 7500 PCR Detection System (Applied Biosystems). The following primer and probe were used: MS2-Forward 5′-GCCTTAGCAGTGCCCTGTCT-3′, MS2-Reverse 5′-AACATGCTCGAGGGCCTTA-3′, MS2-Probe FAM-CCCGTGGGATGCTCCTACATGTCA-TAMRA.

### Molecular Painting

For painting procedures, either concentrated supernatant derived from viral harvesting or medium (DMEM) was mixed with purified GPI anchored protein preparations in 500 μl total volume yielding a final concentration of 35 ng/μl (20 ng/μl for double MP). For M− and V− samples, GPI-anchored proteins were replaced with PSB. For attachment experiments, two sets of samples were prepared and pooled before ultracentrifugation. After incubation for 30 min at 37 °C/5 % CO_2_ under constant agitation, samples were diluted by addition of 36 ml of DMEM and ultracentrifuged as described above. Samples were resuspended in 100 μl or—for attachment experiments—in 500 μl of DMEM for further use.

### Infection and Cytopathic Effects

CrFK and MDCK cells were seeded in 6-well plates and incubated until confluency. Aliquots of 70 μl derived after FMP experiments were used to infect cells. Cells were assessed for cytopathic effects (CPE) after 24 h. Pictures were taken using a Zeiss Axiovert 200 M microscope.

### Pre-treatment of Cells, Adhesion, and Flow Cytometry

HeLa cells were seeded 24 h before infection at a density of 1.5 × 10^6^ cells per well. Two wells of a confluent 6-well plate were used per sample. One hour prior to exposure to virus, cells were either treated with dextrane sulfate (Sigma-Aldrich) to a final concentration of 10 ng/μl or by adding an equivalent amount of virus used for FMP from the same virus stock (taking into account losses by ultracentrifugation and aliquots taken for analysis of the FMP virus, an 2× ratio of blocking to labeled virus was estimated). Modified virus after painting was added to pre-treated or mock-treated HeLa cells, and volume was set to 500 μl. After incubation for 45 min at RT in the dark, supernatant was removed, and cells were washed and scraped into 2 ml of PBS. At this stage, 20 % of the volume was set aside for immunoblot and enzyme-linked immunosorbent assay (ELISA) analysis. Residual cells were inactivated in formaldehyde, washed once in 12 ml of PBS, and analyzed for expression of eGFP in a FACsCalibur flow cytometer (BectonDickinson) using CellQuest software.

### Immunoblotting

Viral supernatants and painting samples were directly mixed with 2× loading buffer (100 mM Tris–HCl, pH 6.8, 20 % glycerol, 5 % SDS, 0.02 % bromo phenol blue) and loaded onto gels. Cell samples were pelleted and treated with lysis buffer (50 mM NaCl, 50 mM Tris–HCl, pH 7.4, 1 % NP40 detergent, 0.5 % sodium de-oxycholate) incubated for at least 30 min at 4 °C before centrifugation at 16,000×*g* for 30 min at 4 °C. Protein concentration of supernatants was measured using a modified Lowry assay (BioRAD Protein DC kit). Samples were separated on pre-cast 4–12 % gradient polyacrylamide gels (Life Technologies) in a 2-(*N*-morpholino)ethanesulfonic acid (MES) buffer system (20× MES buffer: 1 M MES, 1 M Tris base, 69.3 mM SDA, 20.5 mM EDTA, pH 8). After electroblotting (1.1 mA/cm^2^) onto PVDF membranes (Hybond P, GE HealthCare) and blocking, the following primary and secondary antibodies were used: mouse anti-CD59 (Serotec, 1:2,000); mouse anti-HIV-1 p24 (Polymun Scientific, Vienna, 1:2,000); Rabbit anti-GFP (Invitrogen, 1:1,000); rabbit anti-actin (Sigma-Aldrich, 1:1,000). HRP-conjugated anti-rabbit and anti-mouse secondary antibodies were purchased from DakoCytomation and used 1:5,000 (for detection of CD59) and 1:10,000. Signal detection was carried out using the ECLplus kit (GE HealthCare).

### Enzyme-Linked Immuno-Sorbent Assay (ELISA)

A volume corresponding to 10 μg of the protein from samples generated for immunoblot analysis were used for ELISA according to manufacturer’s instructions (CellBiollabs Quick Titer Lentiviral Quantification Kit). Absorbance was measured using a Tecan Genios plate reader.

### Statistical Analysis

All statistical tests carried out were two-tailed, paired Student’s *t* tests using MS-Excel™ for calculation.

## Results and Discussion

### Mono- vs. Dimeric GFP

**Fig. 1:**
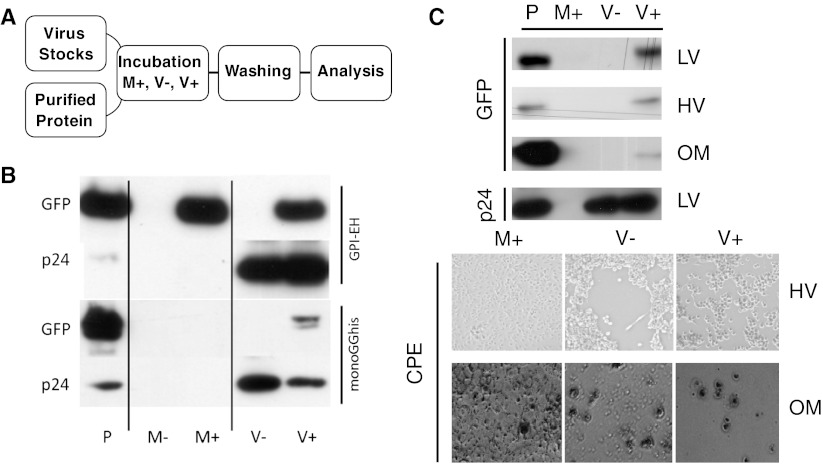
Characterization of fluorescence molecular painting (FMP): **a** schematic representation of MP experiments. Concentrated virus stocks and purified GPI-AP are mixed and incubated. Not associated protein is removed by ultracentrifugation in a washing step. Resulting modified viruses can be analyzed by immunoblot and used for downstream applications. **b** Mono- vs. dimeric GFP-variants. FMP experiments were carried out using a GFP variant prone to induce dimerization at higher concentrations (GPI-EH) and a strictly monomeric version (monoGGhis). A comparable number of lentiviral vector particles were subjected to incubation with the respective GPI-AP. After post-incubation ultracentrifugation, samples were analyzed using immunoblotting with specific antibodies for GFP and p24. Signals were observed in samples containing virus suspension and GPI-AP (V+), as expected. While no signal was present in the M+ washing control when monomeric protein was used, a strong signal in M+ was observed when the dimeric variant was used. All V− samples are negative for GFP. P samples are protein controls for the respective antibodies. Pictures are representative images taken from three independent experiments. **c** Different viral families can be modified with GPI-AP. Concentrated stocks of a lentivirus LV (STAR-A derived), a herpesvirus HV (feline herpesvirus 1, FHV-1), and an orthomyxovirus OM [Influenza A/Aichi/2/68(H3N2)] were incubated with the same amount of GPI-AP and processed as described in **a**. Association was observed for all three virus species, indicated by a signal in the V+ samples, but no signal in the V− and M+ samples. P is protein controls for loading of protein and gauging comparable amounts of protein in the test lanes based on the respective binding of antibodies. Micrographs below show images of CrFK cells infected with FHV-1 and MDCK infected with Influenza A. Pictures were taken 24–48 h post-infection. CPE is clearly visible in samples containing virus particles (V−, V+). M+ control samples show a confluent layer of adherent cells. *M+* medium incubated with GPI-AP during FMP, *V−* virus suspension incubated in the absence of GPI-AP during FMP, *V+* virus suspension incubated with GPI-AP during FMP, *P* purified GPI-AP/p24 control, *LV* lentivirus; *HV* herpesvirus, *OM* orthomyxovirus

Two versions of a GPI-anchored GFP were cloned into an expression vector yielding pGPI-EH and pmonoGGhis, respectively. pGPI-EH encodes an enhanced version of GFP which is prone to dimerization at higher concentrations [23], the gene product of pmonoGGhis remains monomeric even at high concentrations [23]. After stable transfection and expression of monoGGhis and GPI-EH in human embryonic kidney cells (HEK293) and Crandell-Rees feline kidney cells (CrFK), we identified an effective purification strategy to exploit the histidine tag using fast protein liquid chromatography with an immobilized metal affinity chromatography matrix (FPLC/IMAC). In a first set of FMP experiments (see Fig. 1a for a schematic of experimental procedures), both GPI-anchored GFP variants (GPI-EH and monoGGhis) were used to modify lentiviral-like particles produced from STAR cells [25]. For MP, concentrated viral supernatants were incubated with purified GPI-AP for 30 min to 2 h at 37 °C under constant agitation. As controls, virus supernatant was incubated in the absence of GPI protein (V− samples), and cell culture medium containing no virus particles was incubated in the presence of GPI protein (M+ samples). While the first should reveal cross-reactive protein contamination of the viral supernatants, the latter gives information about the efficacy of the post-incubation ultracentrifugation steps in separating viruses with associated protein from not associated GPI proteins. Interestingly, only the monoGGhis protein was shown to give the expected pattern after immunoblotting, i.e., only when virus and GPI anchored proteins were present before ultracentrifugation, is it possible to find a signal after the purification (see Fig. 1b, compare monoGGhis lanes M+ and V+) strongly indicating that the protein is only purified by ultracentrifugation when associated with the enveloped virus. In the case of the dimerization-prone EGFP-based protein, strong signals were also detected in the medium sample (see Fig. 1b, compare GPI-EH lanes M+ and V+). This indicates aggregation of the protein to such an extent as allows the co-purification of protein aggregates with the viral particles: GPI-AP most likely form micelle-like structures in aqueous solutions, due to their amphipathic nature. These micelle-like structures carry multiple copies of the protein, and subsequently, if applicable, multiple copies of the dimer-inducing region on the protein. When in such a situation, two or more of these micelles join via the dimer-forming association; this may be the start of an aggregation event, yielding particles comparable to virus in size and/or sedimentation coefficients, which may co-sediment with the virus during ultra-centrifugation. This suggests that molecules forming di- or multimers may not be suitable for MP, an important aspect for the design of further GPI-anchored proteins for MP applications.

### Different Phylogenetic Groups of Enveloped Viruses are Targets for FMP

In a second set of experiments, three different virus species from phylogenetically diverse families were produced: STAR-A derived lentivirus (HIV-1 based vector, Retroviridae), feline Herpes virus 1 (FHV-1, Herpesviridae), and Influenza A (Influenza A/Aichi/2/68 (H2N3), Orthomyxoviridae). FMP experiments were carried out in a similar way for all three viral species. The most notable difference was the amount of starting material for virus harvesting, to compensate for differences in titers. In all three cases, the expected signal pattern was observed: No signal in the samples containing medium plus GPI-AP (M+) and virus without GPI-AP (V−), but signals in the sample containing both virus and GPI-AP (V+) (see Fig. 1c, compare lanes M+ and V+). To demonstrate presence and infectivity of virus samples after FMP, samples were used for the infection of permissive cells, to then investigate cytopathic effects (CPE). Since LVs do not show a CPE, presence of HIV p24 core protein after painting was used as a surrogate marker for the presence of viral particles. The infectivity of lentiviral particles after painting has been demonstrated previously [12]. The presence of virus was detected in all three cases and the occurrence of CPEs in herpes- and influenza-virus infected cells indicated that virus remained infectious during the procedure (see Fig. 1c, lower panels).

### The Lipophilic Portion of the GPI Anchor is Responsible for Association to Viral Particles

**Fig. 2:**
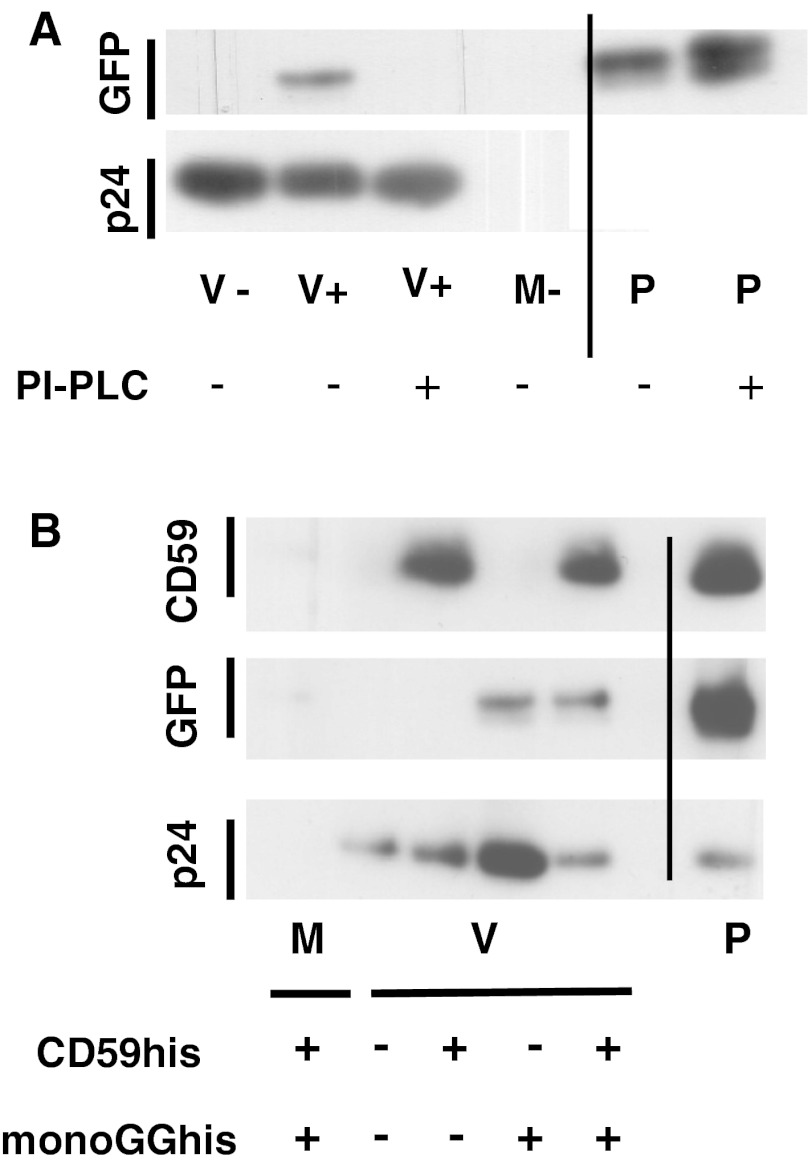
Characterization of fluorescence molecular painting (FMP): **a** molecular painting is dependent on lipid residues in the GPI anchor. MonoGGhis protein containing an intact GPI-anchor was used for FMP, as well as protein pre-treated with PI-PLC, thus rendering it hydrophilic. Comparable amounts of LV virus particles and GPI-AP were used for the experiments. The signal in V+ is lost, when PI-PLC was used to pre-treat the protein. p24 immunoblots indicate the levels of viral particles present. P is protein controls for loading of protein and gauging comparable amounts of protein in the test lanes based on the respective binding of antibodies. **b** Duplex painting. CD59his and monoGGhis were used simultaneously to modify LV particles. No signals were observed for GFP and CD59 in the M+ and V− samples. In the V+ CD59his sample, a signal was only visible in the CD59-specific blot, but not the GFP-specific detection, and vice versa for the V + GFP samples, indicating successful single MP. In V++ signals were detected with both antibodies. No significant difference is seen between signal strength in single and double painted samples. p24 immunoblots indicate the levels of viral particles present. P is protein controls for loading of protein and gauging comparable amounts of protein in the test lanes based on the respective binding of antibodies. *M+* medium incubated with GPI-AP during FMP, *V−* virus suspension incubated in the absence of GPI-AP during FMP, *V+* virus suspension incubated with GPI-AP during FMP, *P* purified GPI-AP/p24 control

Additionally, we were interested to confirm that the GPI anchor alone was responsible for insertion, i.e., not some other mechanism such as non-specific protein–protein interactions. Therefore, cells expressing monoGGhis were treated with phosphoinositol-specific phospholipase C (PI-PLC) to remove the lipophilic parts of the GPI anchor [26, 27]. During this process, the lipophilic moieties remain in the cell membrane. Resulting proteins carry the remnant of the GPI anchor, but not the lipophilic residues. When such a protein preparation was used for FMP of lentiviral particles, the virions did not retain the proteins (see Fig. 2a, compare lanes V+ in the absence or presence of PI-PLC). No signal was found in the V− samples. Again, p24 immunoblots were used to demonstrate the presence of comparable levels of virus in the samples (see Fig. 2a, panel p24). This experiment conclusively shows that the presence of the lipophilic parts of the GPI-AP is required for association with enveloped virus particles.

### Two GPI-APs can be Delivered Simultaneously via FMP

For technical reasons or downstream applications, it may be advantageous if more than one GPI-AP could be associated simultaneously to a sample of viral particles. To test this, purified preparations of both CD59his [12] and MonoGGhis were incubated with a lentiviral vector stock for 30 min. After post-painting ultracentrifugation, resuspended samples were analyzed by immunoblotting using antibodies directed against CD59, GFP, and (again as marker for virus amounts) HIV-1 p24. Control samples containing medium incubated with both GPI-AP (M++) and virus but no GPI-AP (V−) showed no signals, whereas CD59his and monoGGhis single-painted virus samples (V + CD59his and V + monoGGhis, respectively) gave signals only with the respective antibodies. CD59his/monoGGhis double painted samples (V++) gave signals in both “channels” indicating association of both proteins (see Fig. 2b, compare lanes V + CD59his, V + monoGGhis, and V++). At the used concentrations of GPI-AP, no difference in levels of insertion between single and double reactions was observed (see Fig. 2b). However, in preliminary experiments using higher concentrations, both proteins were shown to associate less in the double reaction, most likely indicating competition for available membrane space. Signals for CD59his are generally more pronounced than those for GFP (see Fig. 2b). This may reflect a higher affinity of the antibody, but could also indicate that more molecules of CD59his are associated. Since the molecular weight of CD59his (~18 kDa) is only half of monoGGhis (~35 kDa), it seems reasonable that more smaller molecules may be able to interact with the virus envelope due to decreased steric hindrance.

### Analyzing Attachment Behavior Using FMP

**Fig. 3:**
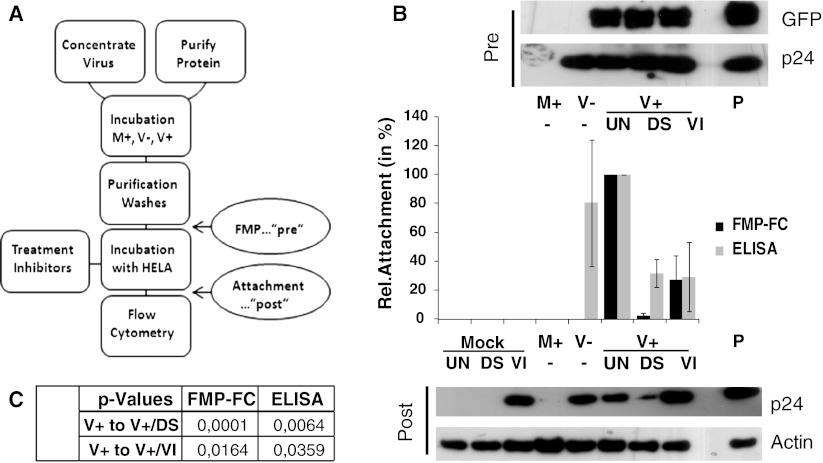
Inhibition of attachment. **a** Schematic representation of the procedure. Lentiviral vectors displaying the amphotropic Env glycoprotein (STAR-A derived) were modified with monoGGhis and incubated with HeLa cells carrying the cognate receptor. Cells were either pre-treated with virus (VI), with dextran sulfate (DS) or left untreated (UN). Before incubation with cells, aliquots of modified virus were analyzed for presence of GFP and p24 (FMP—“pre”-control). After incubation cells were washed and fixed. Subsequently, cells were subjected to flow cytometry to identify cells having virus bound to their surface. In parallel, an aliquot of cells was lysed and used for p24 ELISA and immunoblot, as well as actin immunoblots (Attachment—“post”-control) to confirm viral attachment. **b** After modification of virus particles, the samples were analyzed for the presence of GFP and p24 via immunoblot (Pre). Results indicate successful association, demonstrated by signals in the V+ fractions for GFP, and comparable signal levels for V− and V+ sample in the p24 analysis. In the FMP–FC approach, a clear reduction of attachment levels for the samples treated with inhibitors compared to untreated cells was observed, markedly stronger for DS rather than VI treatment (compare V+/UN to V+/DS and V+/VI). All controls (Mock/UN, Mock/DS, Mock/VI, M+, V−) showed no attachment. In the ELISA approach, similar results were observed. Mock/UN, Mock/DS, Mock/VI, and M+ samples showed no attachment. Attachment was prominent in the V− sample, since the integral viral protein p24 is measured rather than the GFP-label. Reduction of attachment was observed for both inhibitors. For the ELISA, several manipulations were carried out to make data easier accessible: M+, A−, A+ get the Mock value subtracted to set the baseline, Mock/DS, V+/Ds get the Mock/DS subtracted to exclude eventual DS auto-absorption, Mock/VI, V+/VI gets the Mock/VI subtracted, to remove effects from the physical presence of the blocking virus pretreatment. p24 immunoblot of cells incubated with virus (Post) shows the expected pattern: No signals in Mock/UN, Mock/DS, and M+ samples. A strong signal in the Mock+/VI, as a result of the blocking virus, as well as in the V− sample is indicating attachment of the un-labeled virus. V+/DS shows reduced signal strength compared to V+/UN. Potential reduction in signal strength in the V+/VI sample is masked by the presence of the blocking virus. Actin levels suggest that similar levels of protein were used for analysis. P is protein controls for loading of protein and gauging comparable amounts of protein in the test lanes based on the respective binding of antibodies. “Pre”, “Post” immunoblot panels and the attachment data graph are lined up for easier interpretation. **c** Statistical analysis. *P* values generated by using a two-tailed, paired Student’s *t* test are shown, comparing groups V+ to V+/DS and V+/VI for the ELISA and FMP–FC approach, respectively. *M+* medium incubated with GPI-AP during FMP, *V−* virus suspension incubated in the absence of GPI-AP during FMP, *V+* virus suspension incubated with GPI-AP during FMP, *P* purified GPI-AP/p24 control, *Mock* medium not previously used for FMP was used for treating cells, *UN* untreated cells, *DS* dextran sulfate pretreated cells, *VI* virus pretreated cells

Finally, STAR-A produced lentiviral particles modified with monoGGhis were allowed to attach to HeLa cells. Specific binding to the HeLa cells should be mediated by the interaction of the amphotropic murine leukemia virus (MLV) 4070A envelope protein on the virus particles and the cognate cell membrane receptor Pit2 [28]. Cells which had labeled virus attached can be identified by flow cytometry. The complete procedure is summarized in Fig. 3a. First, aliquots of modified STAR-A viral particles were analyzed by immunoblotting using specific antibodies directed against GFP and p24, to assess efficacy of association and levels of virus particles (see Fig. 3b, “Pre” immunoblot panel). To make sure, comparable amounts of virus were used and product-enhanced reverse transcriptase (PERT) assay was performed. The results indicate that the FMP was successful, demonstrated by the appearance of signals in all lanes for samples containing virus incubated with GPI-AP (V+) but not in the M+ or V− lanes containing medium incubated with GPI-AP and virus incubated in the absence of GPI-AP, respectively (see Fig. 3b, “Pre” immunoblot panel). The remaining modified viral particles were incubated with HeLa cells for 45 min at room temperature. A subset of cells was pretreated for an hour either with an excess of lentiviruses (viral samples incubated on such blocking virus pretreated cells are indicated as “VI” in Fig. 3b), thus blocking viral binding sites or dextran sulfate (viral samples incubated on dextran sulfate pretreated cells are indicated as “DS” in Fig. 3b), an efficient inhibitor of lentiviral infection in vitro [29, 30]. Subsequently, the cells were washed, fixed, and prepared for flow cytometry. An aliquot of these cells was lysed and the resulting protein solution analyzed by immunoblotting for p24 and, as an internal control reference, for actin content. Additionally, the protein mix was used for quantifying p24 levels by ELISA found in cells that had been incubated with virus. Since a very high multiplicity of infection (MOI; i.e., the ratio of virus to cell) was used (>10,000 in this case), a 100 % binding rate was assumed for the non-inhibited viral vector, thus all results were set accordingly. Flow cytometry which was undertaken following FMP (FMP–FC) showed a significant decrease in attachment, seen as reduction of cells identifiable by their green fluorescence (see Fig. 3b, central graph, data set FMP–FC, compare Mock, M+ and V+ samples, treated and untreated). Mock samples did receive medium only that had not been subjected to any MP procedure before. ELISA and FMP–FC were complimentary except for two notable differences (see Fig. 3b, central graph). Firstly, in the sample containing unlabeled virus (V−), a prominent signal was detected in the ELISA, but not by the FMP–FC approach. The reason for this is that virus is present in these samples, containing p24 and thus being measurable by ELISA. However, no GPI-AP labeled virions are present in this sample, so a negative result for FMP–FC was expected, indeed acts as a control for unspecific fluorophore contamination and cellular or viral auto-fluorescence. Also, this result actually serves to emphasize the difference between the two methods used. The second discrepancy was seen in the sample containing labeled virus incubated with dextran sulfate pretreated cells (V+/DS) where the relative attachment is considerably higher measured by ELISA than by FMP–FC (see Fig. 3b, central graph, compare V+/DS samples, FMP–FC and ELISA). This is likely due to an unspecific reaction caused by the DS in the ELISA. Also, previous results suggest that a stronger effect can be expected than that which was observed by ELISA, supporting the results from FMP–FC [29]. Immunoblots for actin and p24 were performed to support these findings (see Fig. 3b, “Post” immunoblot panel). Actin blots were used to demonstrate the presence of comparable amounts of total protein. p24 immunoblots showed a decrease in signal strength between untreated cells being incubated with labeled virus to DS pretreated cells being incubated with labeled virus (see Fig. 3b, “Post” immunoblot panel, compare V+/UN and V+/DS). The reduction is masked when comparing samples containing labeled virus incubated either with untreated or virus-pretreated (see Fig. 3b, “Post” immunoblot panel, compare V+/UN to V+/VI) because pretreatment with blocking virus contributes to the total signal strength in this case. Generally, three independent experiments were carried out, and the means and standard deviations were calculated for the combined data. Figure 3c shows a summary table of *P* values derived by using a paired, two-tailed Student’s *t* test. Generally, *P* values were lower for FMP–FC compared to ELISA, probably indicating a higher reliability in this setting. In preliminary experiments using lentivirus-based particles produced by STAR cells lacking the amphotropic Env, we found that approximately 50 % of the attachment level was reached; however, results showed a high variability (48.6 ± 57.4 %; *P* = 0.020). This should indicate the level of unspecific binding at the time of measurement. The high variability observed probably reflects the transient nature of the unspecific bindings but also that normalization between the two virus types using PERT assay may not be optimal. Furthermore, unspecific binding may also explain the relative moderate inhibition of attachment after pre-incubation with virus—this transient unspecific binding would allow for a certain degree of exchange between non-labeled and labeled virus particles.

## Conclusions

We could show that different types of enveloped viruses can be modified with GPI-anchored GFP and that this is absolutely dependent on the presence of the lipophilic moieties of the GPI-APs. Furthermore, we could demonstrate that simultaneous dual surface membrane modification is possible with two different GPI-APs and that FMP can be used to determine the level of deposition of viruses onto cell membranes during the initial steps of infection. Although we believe that labeling viral particles in the described manner has its benefits compared to other membrane labeling techniques (i.e., bio-compatibility), the bigger relevance of MP may be found in other applications. We are currently testing molecules as diverse as streptavidin, CD4, epidermal growth factor (EGF), and interleukin 2 (IL2) for their MP capacities after they have been engineered to contain a GPI molecule post-translationally. The challenge of the approach is in keeping the function of the proteins intact while adding the MP-compatible moiety. Once protein engineering and purification as well as determination of optimal process parameter have been determined for each molecule, the downstream uses are easy to apply. The advantages of the technique are its versatility, flexibility, and speed, combined with a high degree of inherent bio-compatibility (suggesting increased safety for potential use in biomedical or diagnostic applications) and the prospect of delivering multi-functional modifications with duplex/multiplex MP approaches (e.g., a gene delivery vector which is traceable, immuno-protected and targeted). This is most interesting in cases where either different modifications need to be made with the same virus (i.e., infection targeting in gene therapy or immune-modulation of vaccine vectors) or in cases where the same modification has to be applied to a bigger range of virus species (i.e., for labeling or purification). Also, viruses do not need to be cultured meaning that pre-existing, validated systems can be used, and a limited knowledge suffices to introduce modifications whereas other technologies such as antibodies require knowledge of surface antigens in order to target attachment. Interestingly, under certain conditions, MP could also be detected in supernatants from non-virus producing cell lines, most likely indicating association of the GPI-anchored proteins with cell-derived vesicles such as exosomes, which may comprise and additional target for MP.
